# Safety of early oral feeding after total laparoscopic radical gastrectomy for gastric cancer (SOFTLY): Study protocol for a randomized controlled trial

**DOI:** 10.1186/s13063-019-3493-2

**Published:** 2019-06-26

**Authors:** Quan Wang, Bo-Yang Guo, Qing-Chuan Zhao, Zun-Dong Yan, Li-Feng Shang, Juan Yu, Gang Ji

**Affiliations:** Xijing Hospital of Digestive Disease, Xijing Hospital, The fourth Military Medical University, Xi’an, 710032 China

**Keywords:** Gastric cancer, Totally laparoscopic gastrectomy, Early oral feeding, Post-operative fistula, Anastomotic leakage, Enhanced recovery after surgery (ERAS)

## Abstract

**Background:**

Gastric cancer is the third most common cause of cancer-related deaths and has the fifth highest incidence worldwide, especially in eastern Asia, central and Eastern Europe, and South America. Currently, surgery is the only curative treatment for gastric cancer; however, there is an increasing trend toward laparoscopic radical gastrectomy. Early oral feeding (EOF) has been shown to benefit clinical outcomes compared with open gastrectomy under conditions of enhanced recovery after surgery. There are a lack of guidelines and evidence for the safety and feasibility of EOF in patients undergoing laparoscopic radical gastrectomy. Thus, a prospective randomized trial is warranted.

**Methods/design:**

The EOF after total laparoscopic radical gastrectomy (SOFTLY) study is a single-center, parallel-arm, non-inferiority randomized controlled trial which will enroll 200 patients who are pathologically diagnosed with gastric cancer and undergo laparoscopic radical gastrectomy. The primary endpoint, incidence of anastomotic leakage, is based on 1.9% in the control group in the CLASS-01 study. The patients will be randomized (1:1) into two groups: the EOF group will receive a clear liquid diet on post-operative day 1 (POD1) and the delayed oral feeding (DOF) group will receive a clear liquid diet on post-operative day 4 (POD4). The demographic and pathologic characteristics will be recorded. Total and oral nutritional intake, general data, total serum protein, serum albumin, blood glucose, and temperature will be recorded before surgery and at the time of hospitalization. Adverse events will also be recorded. The occurrence of post-operative fistulas, including anastomotic leakage, will be recorded as the main severe post-operative adverse event and represent the primary endpoint.

**Discussion:**

The safety and feasibility of EOF after gastrectomy has not been established. The SOFTLY trial will be the first randomized controlled trial involving total laparoscopic radical gastrectomy, in which the EOF group (POD1) will be compared with the DOF group (POD4). The results of the SOFTLY trial will provide data on the safety and feasibility of EOF after total laparoscopic radical gastrectomy.

**Trial registration:**

Chinese Clinical Trial Registry, ChiCTR-IOR-15007660. Registered on 28 December 2015. The study has full ethical and institutional approval.

**Electronic supplementary material:**

The online version of this article (10.1186/s13063-019-3493-2) contains supplementary material, which is available to authorized users.

## Background

The 2014 World Cancer Report, published by the WHO [1, 2], indicated that gastric cancer was the fifth most common malignancy (951,600 cases; 6.8% of total) and the third leading cause of cancer deaths in 2012 worldwide (723,100 deaths; 8.8% of total). Gastric resection is the main treatment approach by which to prolong the survival of patients with gastric cancer [3].

It has been reported that approximately 30% of cancer patients are malnourished, most of whom are upper gastrointestinal cancer patients [4]. In 1980, the Eastern Cooperative Oncology Group (ECOG) reported that the prevalence of malnutrition among gastric cancer patients was as high as 87% [5].

The disease itself and drug treatment, as well as the effect of surgery on the gastrointestinal tract and metabolic system, are all potential contributors to post-operative malnutrition, which could lead to post-operative infections, prolong the post-operative length of stay (LOS), and increase morbidity and mortality [6–8].

Nutritional support is crucial after gastric surgery. Due to the protection of the anastomotic site and potential transient ileus [9], a fasting period with only parenteral nutrition after gastric surgery until bowel function is clinically detectable is routine [10]. Nevertheless, parenteral nutrition as the only nutritional route has greater nutritional and immunological disadvantages than enteral nutritional support [11, 12].

The European Society for Parenteral and Enteral Nutrition (ESPEN) and Enhanced Recovery After Surgery Society guidelines recommend early initiation of normal food intake or enteral feeding after gastrointestinal surgery (grade A) and enteral tube feeding (e.g., a needle catheter jejunostomy or nasojejunal tube) when oral intake is not possible. Limited data are available regarding immediate oral intake in patients with anastomoses undergoing gastrectomies [13, 14]. Early oral feeding (EOF) as a natural nutritional route after gastric surgery has been recently reported in randomized controlled trials (RCTs) and a meta-analysis, and delayed oral feeding serves as the control [15–19].

These studies have shown an improvement with EOF directly after gastric surgery; specifically, recovery of bowel function was more rapid, the post-operative LOS was shorter, and the surgical and general complication rates were less. Few studies have evaluated EOF in patients with gastric cancer undergoing laparoscopic radical gastrectomy, including total and subtotal gastrectomies [15–18].

Previous studies [15–17] on EOF after open gastrectomy led us to hypothesize that patients with gastric cancer undergoing totally laparoscopic radical gastrectomies treated with EOF have similar or lower anastomotic leakage rates, fewer complications, and a more rapid recovery when compared with delayed oral feeding. Some evidence indicates that EOF as part of early recovery after surgery (ERAS) is feasible and safe after gastric cancer surgery [15, 20] and is associated with a shorter hospitalization and time to gas passage. The surgical procedure and digestive reconstruction are the main differences between open and laparoscopic radical gastrectomy [21] (especially total laparoscopic radical gastrectomy (TLRG)). In the current study, patients with gastric cancer in the EOF group will begin a clear liquid diet on post-operative day (POD)1, while patients in the delayed oral feeding (DOF) group will begin a clear liquid diet on POD4; both groups will have no volume limitations. Only patients undergoing totally laparoscopic gastrectomy will be included. Therefore, we have designed a safety and feasibility RCT to verify the hypothesis. The results may serve as the basis for further study of EOF after TLRG.

The objective of the current study is to assess whether or not EOF after TLRG will increase the anastomotic leakage rate compared with DOF. We seek to determine whether or not a clear liquid diet on POD1 increases the anastomotic leakage rate or could lead to better functional recovery following TLRG compared with oral feeding on POD4. The study design will determine the safety and feasibility of EOF after laparoscopic radical gastrectomy and provide supplementary evidence of ERAS guidelines after TLRG.

## Methods/design

**Fig. 1:**
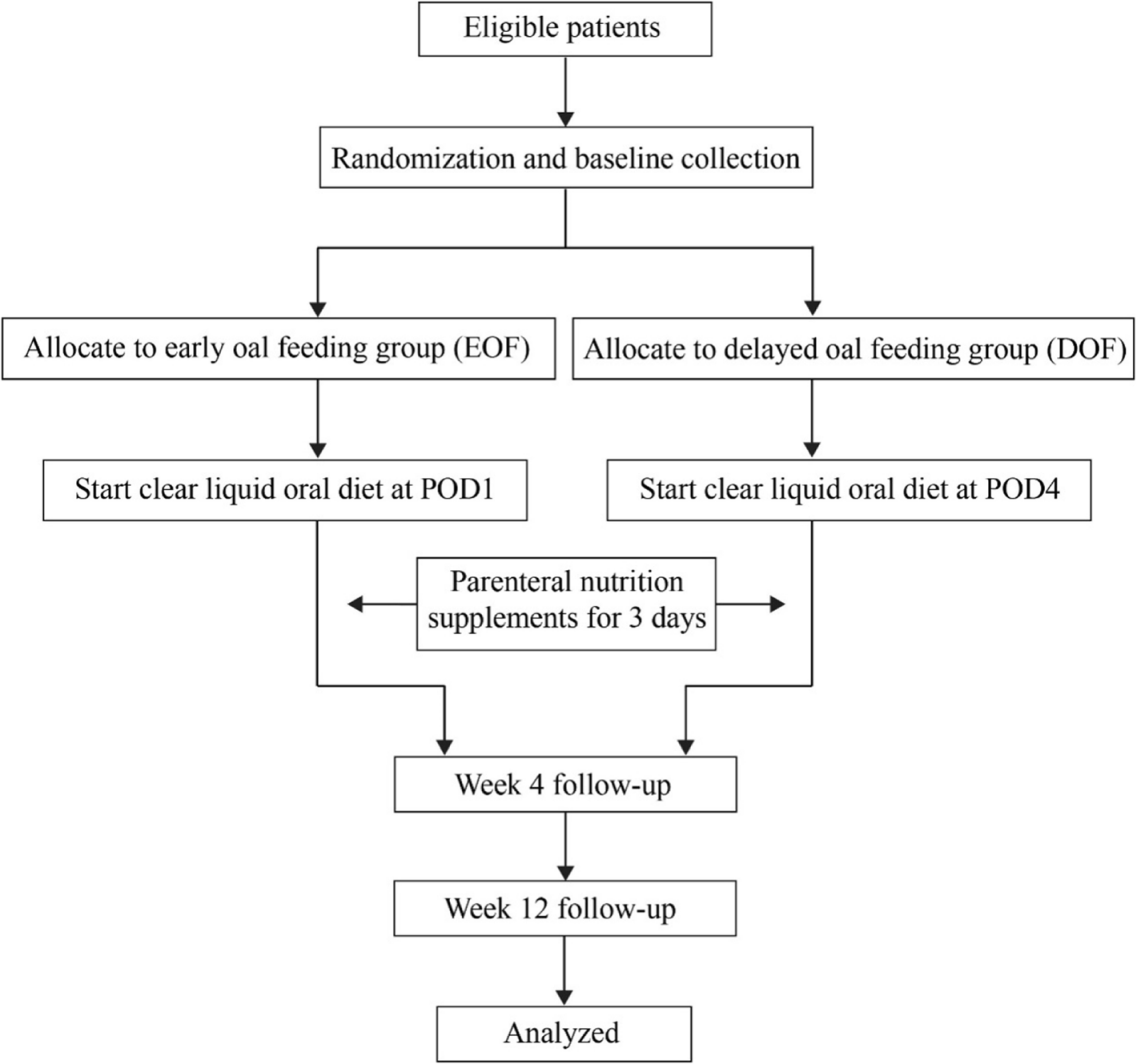
Study design flow chart

SOFTLY is a non-inferiority, single-center, parallel-arm RCT in which 200 patients will be randomly assigned to one of two different times post-operative oral feeding will be initiated after TLRG. The trial flow chart is shown in Fig. 1.

The SOFTLY trial will test the hypothesis that EOF, which will be initiated on POD1, is not inferior to DOF on POD4 for patients undergoing TLRG in terms of anastomotic leakage rate.

A complete checklist of items according to SPIRIT 2013 [22] is provided in Additional file 1.

### Ethics approval

The study procedures and informed consent form have been approved by the independent Ethics Committee of Xijing Hospital in Shaanxi province, China. Information about any adverse events (AEs) will be reported to the Ethics Committee until reaching a stable situation. The Ethics Committee has the duty to periodically evaluate the progress of this trial.

### Randomization procedure

Participants who meet the eligibility criteria will be randomly assigned to either the EOF group or the DOF group with a 1:1 ratio. The sequence of randomization has been generated by a biostatistician who is not involved in this trial with SAS software version 9.2 (SAS Institute, Cary, NC, USA). The randomization list has been sealed in sequentially numbered opaque envelopes, which have been stored in a double-locked cabinet. Randomization is implemented by a research assistant who is not involved in recruitment. After random assignment, the envelopes will again be stored separately. Because the participants cannot be blinded to the intervention or the clinicians responsible for patient care, only data collection and analysis will be blinded [23]. Figure 1 shows the trial flow chart.

### Participant selection

The inclusion criteria are as follows: 1) age range, 18–65 years; 2) diagnosis of gastric cancer and eligible for laparoscopic radical gastrectomy; 3) no chemotherapy, radiotherapy, and targeted therapy before surgery; 4) Nutritional Risk screening 2002 (NRS2002) ≤ 5; and 5) provide informed consent.

The exclusion criteria are as follows: 1) concurrent cancer; 2) remnant gastric cancer; 3) complications (bleeding, perforation, or obstruction); 4) emergency surgery.

### Treatment protocols

This is a confirmatory, single-site, non-inferiority RCT to assess the safety and feasibility of EOF after TLRG compared to DOF.

All eligible patients with gastric cancer will be randomly assigned in a 1:1 allocation ratio to the EOF or DOF group. Participants in both groups will undergo similar peri-operative procedures, with the exception of different oral feeding beginning times. All study data will be stored in an Excel 2007 file, which will be monitored by a nurse who is not involved in this study.

### Peri-operative procedures

Before surgery, gastroscopies, endoscopic biopsies, and computed tomography scans will be performed to confirm the tumor size and location, and patients with organic metastases will be excluded based on assessment by two experienced pathologists. The ERAS guidelines will be followed and all participants will receive pre-operative education, pre-emptive and multimodal analgesia, early ambulation, and laparoscopic radical gastrectomy as part of the peri-operative treatment.

Laparoscopic radical gastrectomies will be performed by the same experienced surgical teams, who have carried out this procedure with an annual caseload of approximately 100 gastric cancer patients. The main anastomosis will be completed laparoscopically, and the abdominal incision will be < 10 cm. After the gastrectomy, Roux-en-Y, Billroth II, or Billroth I reconstruction will be performed in distal gastrectomies and Roux-en-Y esophagojejunostomy in total gastrectomies. Considering the possibility of anastomotic leakage, a peri-anastomotic drain will be placed near the anastomosis after surgery. In most cases, the nasogastric tube will be removed on POD1 and use of a nasojejunal tube will be avoided.

### Intervention protocols

The intervention group will follow the ERAS program and ESPEN guidelines after gastrectomy with respect to enteral nutrition. EOF will be initiated in the form of a clear liquid diet within 24 h after surgery, while a clear liquid diet will be initiated between POD4 and POD6 in the DOF group. In both groups, 1440 mL/day of parenteral nutrition (fat emulsion, amino acids, and glucose injection) or other parenteral nutrition products will be used from POD1–3.

Patients allocated to the EOF group will receive 50–500 mL of 10% glucose injection or other liquid, such as hot pure water on POD1 (within 24 h), followed by 50–500 mL of enteral nutritional suspension on POD2, and > 1000 mL from POD3–6 as tolerated, whereas the participants randomized to the DOF group (control group) will receive the same oral feeding content as described above during this trial (starting on POD4). The only difference between the intervention and control arms will be the oral feeding start time after surgery. Thus, the DOF group will fast for 3 days and receive 50–500 mL of a 10% glucose injection on POD4 and > 1000 mL of enteral nutritional suspension on POD6.

The intervention was planned to POD6. A nutritionist will supervise the oral feeding during the trial using the diet principle of smaller and more frequent intake according to individual tolerance. Adequate caloric intake will be calculated using the Harris-Benedict formula (25–35 kcal/kg/day). An investigator will be assigned to record the clinical observation data.

Only when the gut is intolerant (unable to meet 60% of the daily requirements on POD4) or there is a suspicion of anastomotic leakage, will total parenteral nutrition be considered. Discharge criteria are defined as maintaining normal temperature for 3 days, ambulation, and ability to tolerate oral liquids ad libitum.

### Data collection and schedule

Once informed consent is signed, baseline data, such as age, gender, body mass index (BMI), and complications, will be collected by a clinical research assistant. The laboratory data (total serum protein, serum albumin, and blood glucose) will also be tested pre-operatively and during hospitalization to monitor the patients’ nutritional status. A specified operator will record the details of surgical procedures, such as the operative approach, the location of the tumor, lymph node metastasis, and pathologic TNM stage.

From POD1–6, clinical observation data (anastomotic leakage, the time of first flatus and defecation, NRS pain score, post-operative hospital stay, and complications) will also be recorded daily to evaluate the post-operative recovery by an investigator. The clinicians will be responsible for patient care and will not be involved in data collection.

**Fig. 2:**
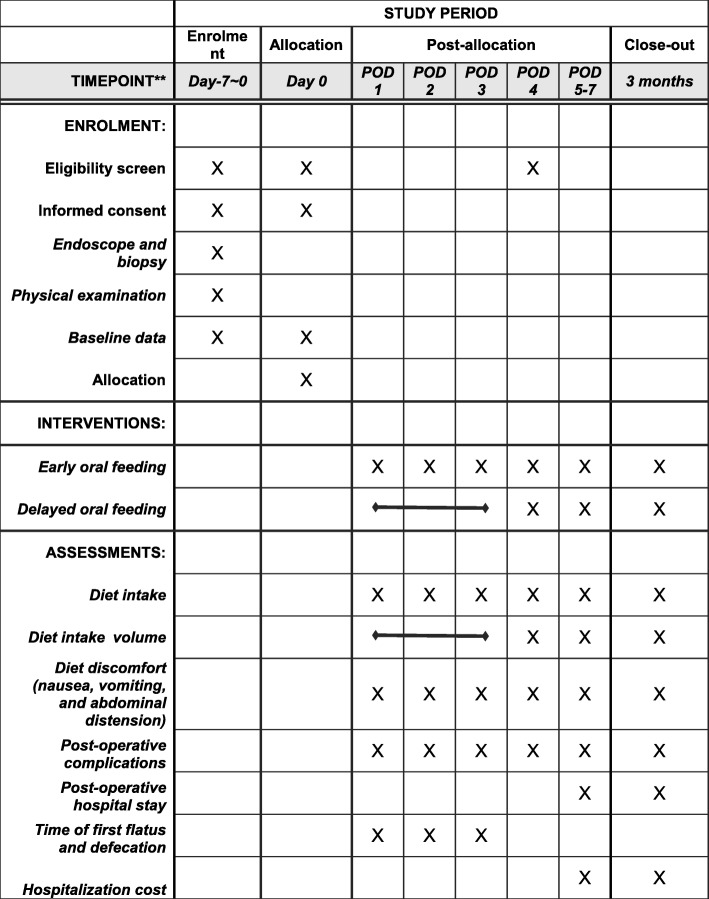
Content for the schedule of enrolment, interventions, and assessments

The contact information and address of patients will be confirmed before hospital discharge. Two follow-up visits will be carried out in the outpatient clinic or via telephone 4 and 12 weeks after hospital discharge, focusing on tolerance of the diet and any discomfort. The details of the schedule are shown in Fig. 2.

### Study endpoints

The primary endpoint will be the percentage incidence of anastomotic leakage after TLRG. We will determine whether or not EOF at POD1 is non-inferior in terms of the occurrence of post-operative fistulas, including anastomotic leakage. Anastomotic leakage will be defined as the breakdown of the connection and subsequent leakage of digestive system fluid from a surgical anastomosis of digestive system structures. If anastomotic leakage is clinically suspected post-operatively, digestive tract radiography will be performed to diagnose the leak. Usually, sufficient abdominal drainage is the most effective treatment.

The secondary endpoints are post-operative recovery and nutritional status, as follows: (1) post-operative complications (n) at 30 days according to the Clavien Dindo classification, which include incisional infection, abdominal abscess, intraperitoneal hemorrhage, anastomotic bleeding, postoperative intestinal obstruction, pancreatitis, pulmonary complications, and others organ complications; (2) the time of first flatus and defecation (day); (3) post-operative length of stay (day); and (4) hospitalization costs (Yuan).

### Sample size estimate

Based on the data from the CLASS-01 study [21], the anastomotic leakage rate in this trial is defined as 1.9%, and previous RCTs involving open gastrectomy concluded that there were no significant differences between EOF and traditional oral feeding (DOF) with regard to the risk for anastomotic leakage [19, 24]. To verify that EOF is non-inferior regarding the anastomotic leakage rate after laparoscopic radical gastrectomy, we designed a non-inferiority test with a non-inferiority margin of 5% (α = 0.05, β = 0.20, and a power of 80%). Considering both clinical and statistical considerations like intolerance of enteral nutritions, therefore, no less than 160 participants (80 participants in each group) will be required. Allowing for a 20% drop-out and withdrawals before trial completion, we decided to recruit a total of 200 participants (100 participants in each group) [21].

### Statistical analysis

The intention-to-treat principle will be applied in all analyses with an assumed drop-out rate. Normally distributed continuous variables will be described as the mean with corresponding SDs, and non-normally distributed or categorical variables will be described as medians with the corresponding range or percentages and frequencies. Normally or non-normally distributed continuous data will be compared by Student’s *t*-test or the Mann–Whitney U test, and the chi-square test and Fisher’s exact test will be used to compare categorical variables, as indicated. With the exception of the primary outcome, secondary outcomes, such as post-operative complications, time of first flatus and defecation, and post-operative length of stay, will also be compared. The potential risk factors for intolerance of EOF and post-operative complications will be identified using a multivariate analysis. A blinded statistician will analyze all of the data using a two-sided *P* value < 0.05 to represent statistical significance.

## Discussion

Factors such as fasting, parenteral nutrition only, stress response after gastrointestinal surgery, and perioperative use of antibiotics can result in inhibition of secretion of saliva and gastrointestinal fluid, impair gastrointestinal motility and intestinal mucosal barrier function, and disrupt the gut microbiome. The use of a traditionally applied nil-by-mouth strategy is often adopted because EOF might lead to a digestive tract fistula or secondary bowel obstruction due to increased intestinal pressure [10, 25]; however, there is no evidence to support this theory. In spite of lacking robust data, the ESPEN and ERAS guidelines after gastrectomy recommend early initiation of oral intake as the preferred route for nutrition.

In recent years, EOF as a nutrition route in accordance with physiological needs after open gastrectomy has been shown to be associated with a decreased hospital LOS without improving the anastomotic leakage rate. Limited data are available for laparoscopic radical gastrectomy.

The proposed study is based on research following open gastrectomy to assess the safety and feasibility of EOF after laparoscopic radical gastrectomy. Under the guidance of ERAS, whether or not EOF will lead to significant benefits will also be assessed compared with DOF.

If EOF after laparoscopic radical gastrectomy proves to be as safe as DOF, additional multicenter studies will be conducted. The effects of content and food intake of EOF on post-operative benefits should be explored in a corollary study, striving to designate EOF as a new nutritional treatment after gastrectomy.

## Trial status

Enrollment for this study is ongoing at the time of manuscript submission. Currently, the trial has already recruited 102 patients.

## Additional file

Additional file 1: SPIRIT 2013 checklist: Recommended items to address in a clinical trial protocol and related documents*. (DOC 115 kb)

## Data Availability

Not applicable.

## Abbreviations

AE: Adverse event
BMI: Body mass index
DOF: Delayed oral feeding
ECOG: Eastern Cooperative Oncology Group
EOF: Early oral feeding
ESPEN: European Society for Parenteral and Enteral Nutrition
LOS: Length of stay
NRS2002: Nutritional Risk Screening 2002
RCT: Randomized controlled trial
